# Application of Ordered Porous Silica Materials in Drug Delivery: A Review

**DOI:** 10.3390/molecules29235713

**Published:** 2024-12-03

**Authors:** Wenwen Liu, Junlin Wu, Zehao Jiang, Xinyu Zhang, Zhenxiang Wang, Fanjun Meng, Zidi Liu, Teng Zhang

**Affiliations:** 1 Nanjing University of Science and Technology Hospital, Nanjing University of Science and Technology, Nanjing 210094, China; 2 School of Chemistry, Chemical Engineering and Materials Science, Shandong Normal University, Jinan 250014, Chinamengfanjun@sdnu.edu.cn (F.M.); 3 Big Data and Intelligence Engineering School, Chongqing College of International Business and Economics, Chongqing 401520, China; 4 Advanced Technology Research Institute (Jinan), Beijing Institute of Technology, Jinan 250307, China

**Keywords:** drug delivery, silica materials, zeolites, ordered mesoporous silica materials

## Abstract

Nanotechnology has significantly advanced various fields, including therapeutic delivery, through the use of nanomaterials as drug carriers. The biocompatibility of ordered porous silica materials makes them promising candidates for drug delivery systems, particularly in the treatment of cancer and other diseases. This review summarizes the use of microporous zeolites and mesoporous silica materials in drug delivery, focusing on their physicochemical properties and applications as drug carriers. Special emphasis is placed on strategies for encapsulation and functionalization, highlighting their role in enhancing drug loading and enabling targeted delivery. In conclusion, while ordered porous silica materials hold great potential for drug delivery systems, certain challenges remain.

## 1. Introduction

Effective drug delivery is crucial for treating a wide range of life-threatening diseases [1,2,3,4]. From traditional small-molecule drugs to modern immunotherapeutic agents, controlled release, targeted delivery, enhanced drug stability, and improved absorption are vital for therapeutic success [5,6]. As advancements in pharmaceutical science and technology continue to progress, the technical requirements for drug delivery systems have also evolved. The development of these systems has kept pace with changing demands, enabling the transformation of various drugs into practical dosage forms [7,8]. However, the emergence of new therapeutic agents, such as peptides, monoclonal antibodies, nucleic acids, and living cells, presents new challenges [9]. These include the stability of peptides, the efficiency of intracellular delivery (e.g., nucleic acids), and the viability and expansion of living cells. Consequently, there is an urgent need for the rapid advancement of drug delivery technologies to accommodate these new agents [8,9].

Ordered porous silica materials, including microporous and mesoporous silica, are of particular interest in drug delivery [8,9,10]. Microporous silica materials, such as zeolites, are crystalline aluminosilicates recognized as promising carriers for controlled drug delivery [9,11]. Zeolites typically have three-dimensional (3D) structures composed of [SiO_4_]^4+^ and [AlO_4_]^5−^ tetrahedra connected by oxygen atoms [12,13,14]. In addition to natural zeolites, various synthetic zeolites have been developed with different Si/Al ratios and heteroatom doping within their frameworks. The pore structure of these zeolites can be modified through various methods, such as post-treatment and templating during synthesis [13,15]. Zeolites also possess excellent ion-exchange capabilities [11,16]. Mesoporous silica materials, including MCM, SBA, MSU, and others, have 3D structures with ordered pores ranging from 2 to 50 nm in size and large surface areas [10,17,18]. The shapes of these mesopores are diverse and can be tailored as needed. Ordered porous silica materials exhibit long-term biological durability, tunability, and versatility, making them suitable for delivering DNA to cells and offering numerous other advantages. Their abundance, high availability, and low cost further contribute to their attractiveness for drug delivery applications [9,10,18,19].

The development of drug delivery systems is expected to significantly enhance drug development and application for several reasons [9,18,19,20,21,22,23,24]: (1) poorly water-soluble drugs can be effectively released, (2) drug molecules can be delivered directly to target cells or tissues, (3) macromolecular therapeutic agents can be encapsulated and released, (4) drugs can cross rigid cell boundaries and tissue barriers, and (5) drugs with varying release rates can be delivered simultaneously to a target location. Therefore, there is an urgent need for continued research and development in drug delivery science, engineering, and technology.

This review focuses on the application of ordered porous silica materials in drug delivery, with a particular emphasis on commonly used zeolites and ordered mesoporous materials. Their advantages in drug delivery are highlighted and discussed in detail.

## 2. Ordered Porous Silica Materials

### 2.1. Zeolites

Zeolites are porous materials composed of silica and aluminum characterized by their three-dimensional skeletal structures [25]. They can be classified into two types: natural zeolites, which form through volcanic activity, and synthetic zeolites [9]. Zeolites are built from TO_4_ tetrahedra, where “T” represents an atom with tetrahedral coordination, such as silicon (Si), aluminum (Al), titanium (Ti), or others [26,27]. Zeolites have been widely applied in various fields, including drug delivery systems [11]. In these systems, pore size plays a crucial role in drug loading and release, making it necessary to adjust the pores for optimal performance. Additionally, zeolites are often modified with various molecules to increase their drug loading capacity and to address the hydrophilicity that exists between the zeolite and the drug [9]. As shown in Table 1, the connection models, channels, tiling arrangements, and cell data of several zeolites frequently used in drug delivery systems are presented.

#### 2.1.1. MFI-Type Zeolites

MFI-type zeolites, such as ZSM-5, possess a microporous structure with elliptical channels measuring 5.5 Å × 5.1 Å along the *a*-axis and 5.6 Å × 5.3 Å along the *b*-axis [14,26,28,29]. ZSM-5, a typical aluminosilicate zeolite of this type, is widely used in drug delivery systems due to its ordered microporous structure and excellent biocompatibility [30,31]. However, other MFI-type zeolites, such as TS-1 and S-1, are less frequently employed in drug delivery [9].

The micropores in ZSM-5 provide space for small-molecule drugs, e.g., gefitinib, which can be loaded onto the zeolite. Drug delivery systems prepared with ZSM-5 have demonstrated excellent drug release and dissolution performance, particularly in targeting A-549 lung cancer cells [32]. However, using ZSM-5 solely as a drug carrier or without modification has significant limitations. To address these limitations, post-treatment methods, such as mesopore creation, and the combination of ZSM-5 with other functional materials are often employed to enhance the effectiveness of drug delivery systems [33].

**Figure 1:**
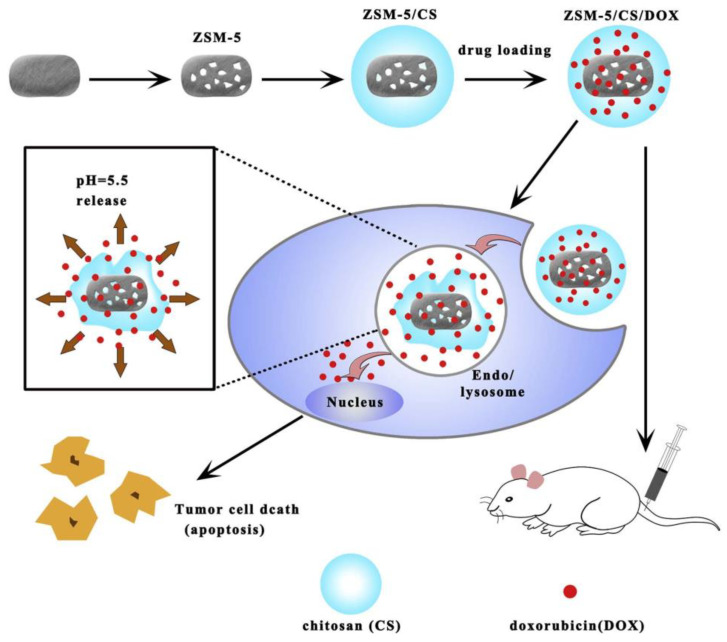
Schematic diagram of the preparation of ZSM/CS/DOX, intracellular DOX release, and DOX distribution [36].

ZSM-5 has also been shown to facilitate mineralized matrix formation [34], and it acts as a drug carrier. However, the diffusion limitations imposed by its microporous structure hinder its application for delivering chemotherapeutic macromolecules [35]. To overcome this, Yang et al. [36] developed mesoporous ZSM-5 that was 300 nm in diameter with 3.75 nm mesopores using an alkaline desilication method. This mesoporous ZSM-5 was coated with chitosan and loaded with doxorubicin (DOX) to create ZSM-5/CS/DOX (as shown in Figure 1). The mesopores provided space for DOX loading, while the chitosan layer regulated its release. The resulting ZSM-5/CS/DOX system exhibited an impressive drug loading efficiency of 97.7% and effectively induced apoptosis in cancer cells for osteosarcoma treatment in vitro evaluation. The CS layer containing numerous amino groups is protonated in the physiological environment at pH 7.4, giving the CS layer a positive charge and thus sealing the mesopores to prevent DOX release. However, when the systems enter the tumor microenvironment, the pH value decreases, leading to deprotonation of the amino groups in the CS layer and loss of the positive charge. Then, the CS layer can no longer seal the mesopores, allowing DOX molecules to be released, and they enter the tumor cells to exert their antitumor effect. In in vivo experiments of treating rats with osteosarcoma, ZSM-5/CS/DOX nanodisks significantly reduced tumor growth and weight compared to free DOX, indicating improved antitumor efficacy. Additionally, the system demonstrated the ability to suppress MG63 cells without systemic toxicity, highlighting its potential in cancer therapy. We believe zeolite ZSM-5 is an idea material for drug delivery systems because of the adjustable structure and the Si/Al ratio. However, the micropores limit their application, and the alkaline desilication will decrease the stability of the zeolite; therefore, the stability of mesoporous ZSM-5 should be paid more attention in its application.

Beyond drug delivery, mesoporous ZSM-5 can also serve as a carrier for functional particles because of its mesoporous structure, chemical stability, and biocompatibility. In a study by Liao et al. [37], mesoporous ZSM-5 prepared through desilication was used to carry NaYF4: Yb^3+^/Tm^3+^ nanocrystals, which acted as a luminescent probe. Doxorubicin was loaded onto the NaYF_4_: Yb^3+^/Tm^3+^@ZSM-5 complex, and its release was accurately monitored using the upconversion emission intensity ratio of I_802nm_/I_475nm_. This drug delivery system exhibited excellent biocompatibility and effectively inhibited tumor cells. The real-time monitoring of the in vivo responsive release process and the mechanism are real challenges, and in the future, more attention should be paid to these challenges.

Recently, drug/polymer electrospun fiber technology has garnered significant interest for drug delivery systems [38]. However, challenges, such as rapid drug release, low mechanical strength, and limited biocompatibility, have restricted its use [31,38]. Fortunately, incorporating zeolite can mitigate these issues [39]. Zarghami et al. [31] combined ZSM-5 with polycaprolactone–polyethylene glycol nanofibers (PCL-PEG NFs) to create a drug delivery system for the sustained release of dexamethasone and ascorbic acid. This combination promoted the differentiation of human-adipose-derived stem cells into osteoblasts. The drug-loaded ZSM-5 significantly reduced the initial burst release, resulting in sustained and prolonged drug release. Experimental results suggest that ZSM-5/nanofiber composites hold promise for developing novel platforms for bone tissue regeneration and engineering.

#### 2.1.2. FAU-Type Zeolite

Faujasite (FAU) zeolite features the skeletal structure of natural octahedral zeolite minerals [40,41,42]. In the FAU zeolite framework, sodalite cages are connected by hexagonal prisms, forming the characteristic structure of the zeolite. The pores in FAU zeolite are arranged perpendicularly to each other, with diameters of 0.74 nm, resulting from 12-membered rings. Each unit cell contains an inner cavity with a diameter of 0.12 nm surrounded by ten sodalite cages [43,44,45,46]. Zeolite Y and zeolite X are typical examples of FAU-type zeolites, with both having aluminosilicate frameworks. Zeolite Y is characterized by a higher Si/Al ratio, while zeolite X has a lower ratio, which is typically less than 1.5 [47,48,49,50].

##### Zeolite Y

Zeolite Y has been explored as a carrier for isoniazid, a potent drug used to treat tuberculosis. Pergher et al. [51] conducted extensive research on the adsorption of isoniazid onto zeolite Y, investigating the effect of pH on adsorption performance. The researchers also performed modeling calculations to study the interaction between isoniazid and zeolite Y during the adsorption process. Faujasite-Y zeolite could change the release profile of isoniazid, but it did not significantly change its release rate; instead, it provided some protective effect, especially in acidic environments, with pH = 3 the zeolite that showed the best performance. Their findings suggest that zeolite Y could serve as an effective delivery carrier for isoniazid, with adjustable drug release properties suitable for antituberculosis treatment. It is worth noting that these research results were only conducted in in vitro release experiments. In in vivo experiments, the effect of Faujasite-Y zeolite on isoniazid may be different.

The use of implantable electrospun nanofibrous materials in drug delivery systems has also been investigated in combination with zeolite Y. Mi et al. [52] loaded curcumin onto zeolite Y with a mean particle diameter of 100 nm to create electrospun nanofibers using a polycaprolactone–gelatin hybrid (curcumin@Y-PG NFs). The inclusion of zeolite Y resulted in steadier drug release over an extended period, i.e., curcumin released from Curc-PG NFs and Curc@nZY-PG NFs was 33.5% and 47% within 72 h and 14 days, respectively, in the in vitro testing. Furthermore, curcumin@Y-PG NFs exhibited excellent anti-migratory activity, cytotoxicity, and pro-apoptotic effects against glioblastoma cells.

**Figure 2:**
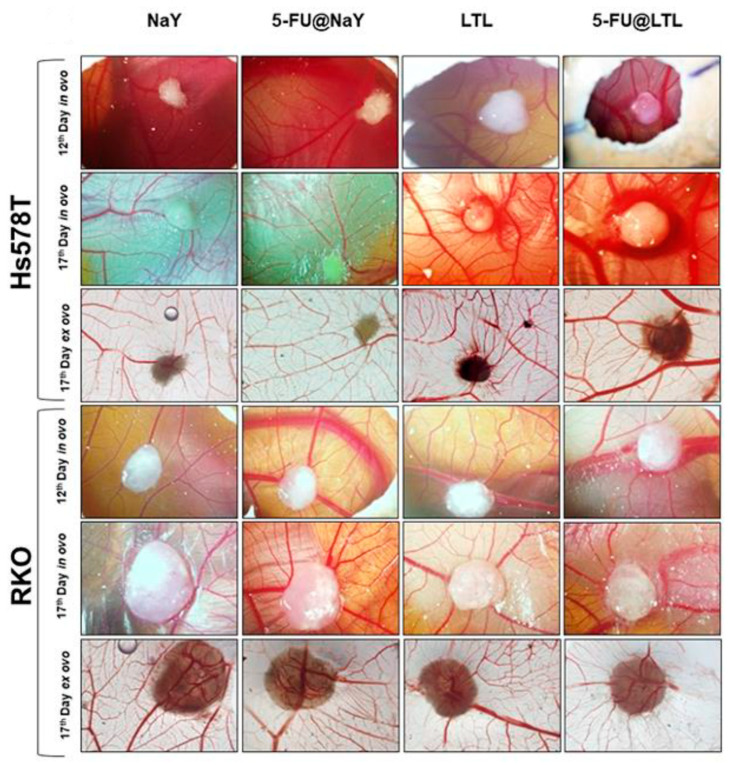
Pictures of tumor growth and angiogenesis on NaY and LTL zeolite in the in vivo study [54].

The framework of zeolite Y provides unique pores for drug loading, as discussed above. Additionally, its exceptional cation exchange capacity offers opportunities for functional modifications. Neves et al. [53] capitalized on these properties by modifying zeolite Y (diameter changed from 100 to 750 nm) with 5-Fluorouracil (encapsulated in the liquid phase) and Ag^+^ (via ion exchange) to create a dual system with antineoplastic and antimicrobial properties. However, the presence of Ag^+^ was found to inhibit the diffusion of 5-Fluorouracil and reduce its loading capacity. Therefore, optimizing the amount of Ag^+^ loaded onto zeolite Y is crucial. The Al element in the framework of zeolite Y should be a very important factor for the adsorption of Ag^+^. Antimicrobial assays demonstrated that both Ag^+^ and 5-Fluorouracil contributed to the antimicrobial activity of the samples, indicating that the prepared dual system holds promise for cancer therapy. In another study by Neves et al. [54], 5-Fluorouracil was loaded onto NaY and LTL zeolite, which was investigated in vivo using a chick embryo chorioallantoic membrane model, as shown in Figure 2. The study revealed that in the zeolite, the main endocytic mechanisms are the caveolin-mediated process and microtubule polymerization for Hs578T and MCF-10 cells.

In another study, Sandomierski et al. [55] utilized the cation exchange capacity of zeolite Y by exchanging Zn^2+^ ions onto it. When ZnY was used as a drug delivery system for 6-mercaptopurine, the drug was effectively trapped on the ZnY surface due to coordination interactions between the drug’s sulfur and nitrogen atoms and the Zn^2+^ ions. The experiments showed that zeolite ZnY could effectively release 6-mercaptopurine. There was no “Burst release” of the drug observed during the first hours, and only 30% of the drug was released from the carriers in the first 10 h, while the rest was released in the following 20 h. It was also found that the drug released without affecting cell viability, highlighting its potential as a biocompatible carrier. Additionally, other cations, such as Ca^2+^ and Mg^2+^, can be ion-exchanged onto zeolite Y and delivered to pre-osteoblastic MC3T3-E1 cells [56]. It was observed that Mg^2+^ and Ca^2+^ were gradually released over 21 days in classical culture media from MgY and CaY, respectively. It should be noted that the excellent ion exchange ability is closely related to the Al in the framework.

**Figure 3:**
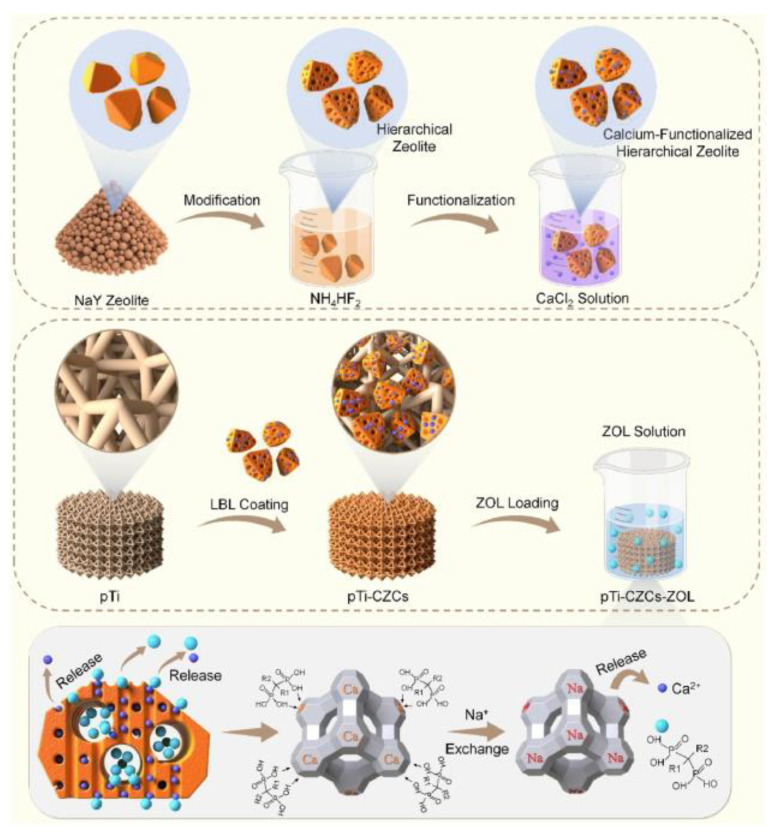
Application of the ZOL-loaded CZCs on the surface of pTi. This system allows for the sequential release of Ca^2+^ and ZOL [59].

Despite the relatively large pore diameter of 0.74 nm in zeolite Y, which is larger than that of other zeolites (e.g., ZSM-5), it still falls within the microporous category [57,58]. To address this limitation, researchers have developed methods to create mesopores in zeolite Y through post-treatment methods. For example, Zhao et al. [59] created mesopores in zeolite NaY by etching it with NH_4_HF_2_ solution. Following Ca^2+^ ion exchange, a Ca^2+^-functionalized hierarchical zeolite Y (CZCs) was obtained. As shown in Figure 3, these CZCs were then integrated into 3D-printed porous titanium alloy implants (pTi), forming pTi-CMCs. This modification increased the zoledronate loading capacity and enhanced the functionality of bone marrow mesenchymal stem cells (BMSCs). The release of Ca^2+^ and zoledronate from pTi-CMCs significantly promoted osteogenesis. The unique structure of pTi-CMCs helped regulate the osteogenic–osteoclastic balance. In order to test the biocompatibility of the constructed drug delivery systems in vivo, subcutaneous implantation in rabbits was carried out, with no significant pathological effects on the liver, heart, spleen, kidneys, or lungs for two weeks. All of the experiments suggested that prosthetic implants using this approach could be effective in managing osteoporosis. However, we believe it should be noted that the post-treatment of zeolite Y definitely destroyed its framework, which means the stability of the zeolite decreases, and the Al element or some nanoparticles will be easily released from the drug delivery systems. So, the above side effect should be considered in real applications.

##### Zeolite X

Similarly to zeolite Y, zeolite X also possesses the FAU framework structure [60,61]. However, zeolite X is distinguished by its lower Si/Al ratio, which ranges from 1 to 1.5. This lower ratio results in a higher cation exchange capacity and a stronger adsorption capacity due to the increased negative charge in the framework [62,63].

It is well-known that some active pharmaceutical compounds suffer from poor solubility, limited permeability, and low bioavailability when administered orally [64,65,66]. Zeolite X, a typical microporous zeolite, has been employed in oral drug delivery systems to enhance the solubility of poorly water-soluble compounds. For instance, Fatouros et al. [67] utilized an incipient wetness method to load danazol onto microporous zeolite X for oral delivery. Even under accelerated storage conditions for six months, the drug payload and stability remained high. In the ex vivo assessment, the relative permeability of danazol from NaX was twice that of crystalline danazol recorded at 30 min (*t*-test, *p* < 0.05). The work demonstrated that microporous zeolite X could be a promising material for the oral delivery of lipophilic drugs like danazol with poor solubility.

Researchers have also explored the development of mesoporous zeolite-X-based drug delivery systems. In the study by Binay et al. [68], intracrystalline voids were created using a post-treatment method to obtain hierarchical zeolite X. When only thymol was loaded onto zeolite X, no antimicrobial activity against Staphylococcus aureus was observed. However, when Zn^2+^ was loaded onto zeolite X, thymol was loaded onto hierarchical zeolite X, or both thymol and Zn^2+^ were loaded onto hierarchical zeolite X, significant antimicrobial activity was detected. The results indicated that both Zn^2+^ loading and the hierarchical structure influenced the release profiles, contributing to enhanced antibacterial activity and stability through controlled release. In zeolite X, the content of Al in the framework is a little higher than in zeolite Y and ZSM-5; therefore, the stability of the zeolite should attract more attention.

Given the similarities between zeolite X and zeolite Y, Sandomierski et al. [55] compared the two as carriers for 6-mercaptopurine. Zn^2+^ was ion-exchanged onto the frameworks of both zeolites, and 6-mercaptopurine was subsequently trapped on the surface through coordination interactions with Zn^2+^. Due to the higher aluminum content in the framework of zeolite X, more Zn^2+^ ions were exchanged, leading to a stronger interaction with 6-mercaptopurine and potential pore clogging. This study highlights that the Si/Al ratio in FAU-type zeolites significantly affects the performance of drug delivery systems.

Hydroxychloroquine, an active pharmaceutical ingredient used in the treatment of COVID-19, has also been anchored onto zeolite NaX for targeted delivery within the human body [69]. In a study by Goscianska et al. [69], zeolite NaX with diameters of ca. 6 μm was prepared using a low-cost method. It was found that the adsorption process was aligned with the Freundlich isotherm model, with a high adsorption capacity of 114 mg/g. The particle size, textural parameters, morphology, and chemical composition greatly affected the drug’s release. Additionally, under conditions mimicking intestinal fluid (pH = 5.8), zeolite NaX effectively regulated the release of hydroxychloroquine.

Zeolite NaX has also been employed in drug delivery systems for doxorubicin [70]. In this approach, NaX and doxorubicin were simultaneously incorporated into a solution of poly (lactic acid) and chitosan to fabricate poly (lactic acid)/chitosan/NaX/Fe_3_O_4_/doxorubicin nanofibers via electrospinning. These synthesized nanozeolites were then used for the controlled release of doxorubicin to induce carcinoma cell death.

#### 2.1.3. LTA-Type Zeolite

LTA-type zeolite is an eight-membered ring molecular sieve with a pore diameter of 4.2 Å. Its octagonal three-dimensional structure consists of eight β-cages at the vertices of the crystal cell, which are interconnected to form larger α-cages. Adjacent α-cages are linked to create the main pore system [71,72,73,74].

Zeolite type A, one of the earliest and most extensively studied molecular sieves, possesses an LTA topology and is a key representative of microporous molecular sieves [75,76]. The pore size of zeolite type A can be adjusted by varying the size and charge of the cations, ranging between 3.0 and 5.0 Å [77]. Specifically, the pore sizes for K-A (3A), Na-A (4A), and Ca-A (5A) zeolites are 3.0 Å, 3.8 Å, and 4.8 Å, respectively [78,79,80,81]. Neves et al. [82] explored the use of NaA (with a particle diameter of ca. 5 μm according to the SEM images) zeolite as a host for the delivery of α-cyano-4-hydroxycinnamic acid (CHC), an experimental anticancer drug. CHC was loaded into the zeolite’s porous structure via liquid-phase diffusion. The impact of NaA and CHC@NaA on the viability of the HCT-15 human colon carcinoma cell line was evaluated, revealing that NaA itself was non-toxic to the cancer cells. Remarkably, CHC@NaA demonstrated a 585-fold increase in cell viability inhibition compared to the unencapsulated drug, highlighting the significant potential of NaA zeolite for drug loading and delivery in cancer treatment.

In another study by Abukhadra et al. [83], zeolite-A with an average diameter of 2.3 μm was functionalized with β-cyclodextrin (β-CD/ZA) and used in drug delivery systems for levofloxacin (LVC), enhancing its loading, release, and anti-inflammatory properties. Functionalization with β-cyclodextrin increased the active site density, leading to a high LVC loading capacity of 363.4 mg/g. Advanced equilibrium studies indicated that LVC was loaded onto β-CD/ZA through a combination of physical and multimolecular mechanisms, with adsorption following pseudo-first-order kinetics and exhibiting homogeneous, monolayer loading properties. The loading process was spontaneous and exothermic. LVC was released from β-CD/ZA in a prolonged, slow, and continuous manner over 200 h in the in vitro release study. Furthermore, the prepared β-CD/ZA demonstrated enhanced anti-inflammatory effects by reducing cytokine (IL-6 and IL-8) production in human bronchial epithelial cells (NL20).

Recent investigations into the use of LTA-type zeolite in drug delivery have been limited, likely due to the small diameter of its channels. These small channels restrict the application of LTA-type zeolite for drug delivery, as many drugs have larger molecular structures than the channels can accommodate. As suggested in other studies, creating mesopores in LTA-type zeolites could potentially expand their applicability for a wider range of drugs in delivery systems [9,18].

#### 2.1.4. Beta-Type Zeolite

Beta-type zeolite, a member of the intergrowth family, is a macroporous molecular sieve characterized by a three-dimensional, twelve-membered ring pore structure, offering a more open pore system compared to MFI, MOR, and MWW zeolites [9,58,84]. Beta zeolite consists of two distinct polymorphs, A and B, which grow as 2D sheets that randomly alternate, forming a 3D network of 12-ring pores [85]. While the intergrowth of these polymorphs does not significantly affect the pore size, it can cause the pores to become tortuous in the direction of the faulting, although without leading to blockage [9].

Due to the large micropores in beta zeolite, it is capable of adsorbing drug molecules within its pores. For instance, Souza et al. [86] used beta zeolite as a carrier for isoniazid, a first-line drug for treating tuberculosis. In vitro release kinetics were studied in both an acidic dissolution medium (pH = 3) and simulated intestinal fluid (pH = 6.8) without enzymes, mimicking the release profiles in the gastrointestinal tract. The study showed that the majority of the drug was adsorbed into the micropores of the zeolite, with a retention capacity of about 110 mg of drug per gram of zeolite. The release kinetics indicated that drug release was more sustained in acidic conditions compared to intestinal fluid.

The excellent loading capacity of beta zeolite allows it to simultaneously carry multiple drugs. For example, in a study by Szegedi et al. [87], beta zeolite aggregates of 150–500 nm in diameter were loaded with silver using a mechanochemical method, followed by the deposition of the sulfadiazine (SD) drug. Characterization results confirmed the incorporation of Ag ions into the zeolite framework through solid-state ion exchange. Their experiments demonstrated the ability of beta zeolite to store and release both SD and Ag ions simultaneously. It should be noted that the adsorption of positive cations has a close relationship with the Al in framework of beta zeolite. In vitro release studies at pH = 5.5 revealed that the presence of both drugs influenced their release kinetics. In the systems, about 44% of Ag was released with the release of 20% SD within 8 h. When tested in various bacterial strain models, the presence of Ag ions enhanced the bactericidal effect of the drug delivery system.

While the micropores of beta zeolite play a critical role in drug delivery, the surface area of the zeolite is also significant in some drug delivery systems. In the work of Fatouros et al. [88], a co-spray drying method was used to load nifedipine (NIF), a BCS Class II drug, onto beta zeolite. The study found nearly 100% encapsulation efficiency, with both the pore network and the surface area contributing to the effective drug loading. In simulated gastric (pH = 1.2) and intestinal fluids (pH = 6.8), the in vitro dissolution rate of NIF was significantly increased, i.e., the pure drug dissolved was 18%; however, the drug release amount was 6-fold for the system in 15 min. This research highlights the potential for enhancing the dissolution of poorly soluble drugs, thereby improving their oral bioavailability.

#### 2.1.5. HEU-Type Zeolite

Unlike the previously mentioned zeolites, HEU-type zeolite is a natural form of zeolite [89]. Due to its unique properties, such as its cost-effectiveness, low toxicity, abundance in nature, and high environmental friendliness, natural zeolite has garnered significant attention across various fields, despite often containing impurities [90]. HEU-type zeolite features a microporous tetrahedral arrangement of silica and alumina, with structural properties outlined in Table 1. HEU-type zeolites typically include two varieties: clinoptilolite and heulandite [89,91]. In clinoptilolite, the Si/Al ratio in the framework is ≥4, whereas in heulandite, the ratio is <4 [91].

**Figure 4:**
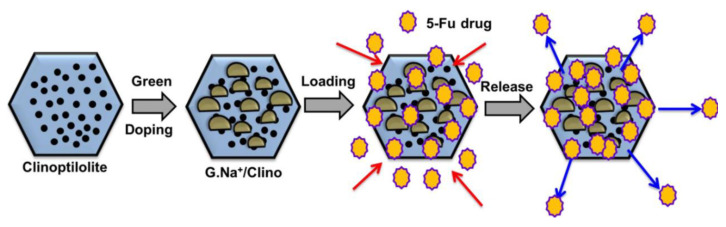
The scheme of Na^+^/clinoptilolite preparation and application in the drug delivery systems [90].

Clinoptilolite has been used in drug delivery systems. Abukhadra et al. [90] prepared Na^+^-functionalized clinoptilolite using a green method and applied it for the delivery of 5-fluorouracil (5-FU), as shown in Figure 4. This system improved the application of 5-FU in treating various cancers, including rectal, breast, and stomach cancers, by overcoming its challenges related to limited solubility, low selectivity, a high diffusion rate, and toxicity at high doses. The study demonstrated that the drug loading capacity of the carriers was high and controllable (the theoretical loading capacity could reach 462.7 mg/g), and drug release occurred in a controlled and sustained manner, lasting up to 150 h at pH 1.2 and 80 h at pH 7.4. This system enhanced the cytotoxic effect of 5-FU in colon cancer treatment. In a similar study, Abukhadra et al. [92] modified clinoptilolite with MgO nanoparticles instead of Na^+^ (G. MgO/Clino). After the modification, the loading capacity for 5-FU increased from 163 mg/g to 244.5 mg/g. A long and continuous profile in the release study was obtained, i.e., 150 h either in gastric buffer (pH 1.2) or intestinal buffer (pH 7.4). In addition, the in vitro cytotoxicity study on the colorectal normal cell (CCD-18Co) indicated that the drug delivery system was both safe for and biocompatible with normal colorectal cells.

In a study by Kukobat et al. [93], the anticancer drug letrozole (LTZ) was directly adsorbed onto clinoptilolite with particle diameters of 10–20 µm. Rapid in vitro dissolution of LTZ was observed, reaching nearly 80% after 1 h of dissolution, which was attributed to its uniform exposure on the external surface of the zeolites. The small positive adsorption energy value (0.06 eV) indicated favorable release in aqueous media. Although the authors suggested that clinoptilolite might be mesoporous, they did not explain how these mesopores formed. We hypothesize that the mesopores resulted from the HCl treatment during surface activation, leading to intercrystalline mesopores. In addition, the large diameter of the zeolite should be worth noting.

To enhance drug delivery performance, modifying clinoptilolite is an effective strategy. In a study by Dragan et al. [94], chitosan was grafted onto clinoptilolite using cryogelation, and the resulting composite was applied to drug delivery systems for diclofenac sodium (DS) and indomethacin (IDM). The ratio of chitosan to clinoptilolite was critical in constructing the porous architecture. The chitosan/clinoptilolite composite demonstrated a rapid response across the pH range of 1.2 to 7.4, and it exhibited excellent stability. The study showed that the prepared composites were promising materials for drug delivery in the intestinal tract.

#### 2.1.6. MOR-Type Zeolite

Mordenite (MOR) is one of the most abundant natural zeolites found in altered volcanic deposits and marine sediments [9,95]. Its molecular structure consists of five-membered rings of silicate and aluminate tetrahedrons, where four oxygen atoms form a triangular pyramid around a central silicon or aluminum atom. MOR-type zeolites are highly resistant to acid due to their high silicon-to-aluminum ratio [9,96].

Kusuma et al. [97] applied natural MOR zeolite in drug delivery systems. To address the presence of impurities in natural MOR, a purification process using reverse osmosis was conducted. The MOR zeolite was then modified with Cu(II) to create a new material suitable for safe oral drug delivery of ibuprofen and meloxicam. The release of meloxicam and ibuprofen from Cu(II)-Mor presented the highest release at 1200 min in pH 7 with a dissolution percentage of 93.17% and 90.98%, respectively. The study observed excellent cytocompatibility, drug loading capacity, controlled release, and stability in the prepared composite, highlighting its potential for oral drug delivery applications. Additionally, the researchers prepared Zn-modified natural MOR zeolites for ibuprofen delivery [98]. Performance evaluations, including drug loading (content 90.37%) and release (at 1080 min and pH 7 with a current dissolution of 96.747%), zeta potential measurement (−61.55 ± 0.1 mV), and cytotoxicity testing, demonstrated that Zn/MOR zeolites are promising alternatives for drug delivery systems. However, the stability of Zn/MOR should be studied deeply in in vivo studies, because the Zn cations may also release from the drug delivery systems, thus affecting the cells.

### 2.2. Ordered Mesoporous Silica Materials

Ordered mesoporous silica materials (MSMs) typically feature continuously adjustable mesopores with diameters ranging from 2 to 50 nm [99,100]. As novel inorganic materials, MSMs possess highly ordered mesoporous structures, exceptional surface areas and pore volumes, favorable biocompatibility, and hydroxyl groups on their surfaces [99,101]. These characteristics make MSMs ideal for use in drug delivery systems.

#### 2.2.1. MCM-41

MCM-41, a type of MSM, has uniform one-dimensional pores with a narrow size distribution adjustable between 2 and 10 nm [102,103]. This design gives MCM-41 a high adsorption capacity and favorable diffusion properties for organic molecules, making it highly effective in drug delivery systems [10,18,104].

Due to its unique properties, MCM-41 can perform well in drug delivery systems, even without modifications. For instance, bicalutamide (BLT), an anticancer drug, can be loaded onto MCM-41 with a particle diameter of ca. 412 nm using a solvent impregnation method [105]. This process allows for a drug loading capacity of up to 40%, resulting in a reduced initial burst effect and slower drug release compared to the pure drug. Compared with the zeolite with micropores, MCM-41 has greater drug loading due to its unique structure. Additionally, MCM-41 demonstrates excellent blood biocompatibility. The incorporation of BLT into MCM-41 enhances its in vitro antitumor activity against prostate adenocarcinoma LNCaP cells. Researchers have also explored dual drug loading on MCM-41 to exploit synergistic interactions and improve efficacy against resistant bacteria. For example, Oliveira et al. [106] developed an Ofloxacin@Doxorubicin–Epirubicin-functionalized MCM-41 to investigate its antibacterial activity. The study indicated that the system was pH-sensitive due to the fact that the percentage of drugs released from the particles at pH 4 was higher than at pH 7.4 in the in vitro study. The work revealed the potential of MCM-41 in versatile drug delivery applications. However, it should be noted that the stability of MCM-41 is inferior to the zeolite without post-treatment.

To further enhance drug delivery performance, MCM-41 can be combined with other materials. Weng et al. [107] integrated cobalt ferrites–oleic acid (CF-OA) with MCM-41 for pH-responsive doxorubicin delivery. Their study examined the effects of temperature, nanostructure, and drug release kinetics, contributing to the development of more stable and effective drug delivery systems. Sohrabnezhad et al. [108] prepared a polyimide-COF/amino-functionalized MCM-41 nanohybrid for erythromycin (E) antibiotic delivery, demonstrating good biocompatibility and potential for drug delivery applications. Additionally, *tert*-Butylamine (TBA)-functionalized MCM-41 showed exceptional performance in controlled release systems for the anticancer drug cyclophosphamide [109]. Specifically, with 17% TBA modification, MCM-41 exhibited optimal performance in drug loading and release.

#### 2.2.2. SBA-15

SBA-15 is a well-known mesoporous silica material with a hexagonal structure. It features high surface areas, large pore volumes, excellent pore wall thickness, and good thermal stability [110,111]. To enhance the drug adsorption and release capabilities of SBA-15, researchers often modify it with various functional groups [112,113].

For example, Choi et al. [112] functionalized SBA-15 with amidoxime (AMI) to create SBA-15@AMI nanoparticles. This modification added a substantial amount of AMI to SBA-15 (which was 150 nm in diameter) without altering its morphology. Doxorubicin hydrochloride (Dox), an anticancer drug, was loaded onto SBA-15@AMI nanoparticles, demonstrating the carriers’ ability to load drugs and exhibit pH-responsive release behavior. In the in vitro release of DOX, at pH 7.4, 23% of the DOX was released within 24 h; however, the percentages were 78% and 92% at a pH of 6.5 and 5.0, respectively. In another study, Albayati et al. [113] prepared NH_2_-SBA-15 and used it as a sorbent for drug loading from solutions. Their experiments showed that the amino group modification significantly enhanced the drug loading capacity. Drug adsorption on SBA-15 (with a particle size of ca. 100 nm) occurs through hydrogen bonding between the drug and the silanol groups of SBA-15, as well as through physical interactions with the functional groups on SBA-15. In further research, curcumin (CUR) was encapsulated in NH_2_/SBA-15 to produce CUR@NH_2_/SBA, which was used in an effective CUR delivery system. Both the loading efficiency (89.7%) and the release behavior (41.2% release after 72 h) of CUR in a phosphate buffer solution at pH 7.4 were evaluated, showing that NH_2_/SBA-15 is an excellent carrier for CUR loading and controlled release. Prokopowicz and Szewczyk [114] investigated NH_2_/SBA-15 prepared via a post-synthesis grafting method for cefazolin delivery systems. Their research demonstrated prolonged drug release up to 7 days and mineralization properties with delayed hydroxycarbonate apatite formation.

#### 2.2.3. MCM-48

MCM-48 features a three-dimensional (3D) cubic Ia$\bar{3}$d mesoporous structure consisting of two interpenetrating networks of chiral channels [115]. Unlike MCM-41, which has a 2D hexagonal structure, MCM-48’s 3D channel network provides highly open porous hosts that facilitate the inclusion and diffusion of guest molecules [116,117]. These properties make MCM-48 suitable for drug delivery systems [118,119].

Like other mesoporous materials, MCM-48 is often modified to enhance its functionality. Patel et al. [118] functionalized MCM-48 (260–265 nm in particle diameters) with 12-tungstophosphoric acid (TPA) for controlled doxorubicin hydrochloride (DOX) release. Their study showed that the drug concentration remained stable throughout the release process. The in vitro release study was investigated in simulated body fluid (pH 7.4, 37 °C). The DOX release increased from 7% to 62% after 8 h from DOX/TPA/nMCM-48, and after 24 h, the value increased to 90%. In the in vivo study, the system was assessed through the induction of liver cancer on a nude mouse model, and the results demonstrated the excellent performance of DOX/TPA/nMCM-48 in delivering DOX and the application of a good treatment regimen. In addition, the study indicated that the MCM-48-based delivery system was non-toxic and effective in tumor regression. Meléndez-Ortiz et al. [119] grafted MCM-48 with polyacrylamide (PAAm) using an azo-type initiator. This modification did not alter the morphology or mesopore structure of MCM-48. In nalidixic acid delivery systems, the drug loading on MCM-48-PAAm was significantly higher compared to unmodified MCM-48, with a sustainable release of approximately 80%. Zelenák et al. [120] further demonstrated that functionalized MCM-48 outperforms pure MCM-48 in drug delivery systems. MCM-48 with a particle size of 90–120 nm was modified with 3-aminopropyl groups to serve as carriers for the poorly soluble anti-inflammatory drug indomethacin. Post-modification, the indomethacin loading increased from 21% to 45% by weight, and its release rate was slower. The pH of simulated body fluids also influenced the release amount of indomethacin.

In addition to functional group modifications, some researchers have combined MCM-48 with other materials for drug delivery systems. Pajchel and Kolodziejski [121] coated MCM-48 (with a diameter of 210–240 nm) with nanohydroxyapatite (nano-HA) to create a novel drug delivery system. The nano-HA coating, applied using silanols, did not block the mesopores of MCM-48. The MCM-48/nano-HA composite retained excellent adsorption properties for ibuprofen (Ibu), which was incorporated into the mesopores. This study highlighted the potential of the MCM-48/nano-HA composite for use in drug delivery systems.

#### 2.2.4. SBA-16

The mesoporous silica material SBA-16 (Santa Barbara Amorphous-16) features a cubic structure with the space group Im3m, characterized by two non-interpenetrating 3D channel systems connected by spherical cavities [122,123]. Due to its high surface area and unique structure, SBA-16 has been widely utilized in various applications [124].

SBA-16 is often compared to SBA-15. For example, Gonzalez et al. [125] conducted a comparative study of SBA-15 and SBA-16 in clindamycin delivery systems. Both materials were coated with apatite, and their drug delivery performance was evaluated under different pH conditions. The study found that both SBA-15 and SBA-16 showed promise for clindamycin delivery. However, SBA-15 outperformed SBA-16 in terms of bioactivity in simulated body fluid and clindamycin release. This improved performance is attributed to the differing textural properties of the two materials. The authors concluded that SBA-15 is more suitable as an osteogenic material, promoting mineralization nodule formation.

Other studies have explored the application of SBA-16 in drug delivery systems. For instance, SBA-16 was modified with aluminum (SBA-16-Al) for use in the adsorption and release of rifampicin, a drug used to treat tuberculosis [126]. Modification with aluminum increased the surface area of SBA-16, i.e., the surface areas of SBA-16 and SBA-16-Al were 624.3 and 843.5 m^2^/g, respectively. In the in vitro simulation study, during the first 8 h, the release of rifampicin from SBA-16-Al was slow, as expected. However, during the time of 8–9 h, a brush effect was observed. Rifampicin adsorption on SBA-16-Al was found to be heterogeneous, non-spontaneous, and stable. The release kinetics of rifampicin followed the Higuchi model. However, the biocompatibility of SBA-16-Al was not assessed in this study.

Additionally, SBA-16 has been used to encapsulate ZnO nanoparticles, forming ZnO/SBA-16, which was applied in temozolomide delivery systems [127]. The results demonstrated that SBA-16, after loading with ZnO, exhibited controlled drug release. Martines et al. [128] also showed that rutin could be loaded onto SBA-16, producing Rutin-SBA-16, which proved to be a promising drug delivery system.

## 3. Concluding Remarks: Challenges and Future Directions

### 3.1. Challenges

Drug Release Control: One major challenge is controlling drug release. Small pore sizes can limit the loading capacity, especially for drugs with large molecular volumes. To address this, silica materials need to have pores with an appropriate size to facilitate both adsorption and release. This often requires modifying their textural characteristics through various methods. For instance, mesopores are typically created in microporous zeolites using post-treatment methods, although mesoporous silica materials can sometimes be used directly. While ordered mesoporous silica materials can overcome issues related to small pore sizes, they still face challenges, such as low stability and reproducibility on an industrial scale.

Toxicity Concerns: Some silica materials may have cytotoxic and carcinogenic effects. For example, erionite, a brittle and wool-like fibrous zeolite, has been linked to lung cancer and malignant mesothelioma. Offretite and skolecite may also exhibit cytotoxicity, disrupting the cell’s structure and leading to swollen mitochondria and squared cells. Zeolite NaA can interfere with mineral metabolism and tissue mineral composition, with both aluminum and silicon elements potentially penetrating body tissues. Therefore, the toxicity of ordered porous silica materials must be carefully considered when used in drug delivery systems.

### 3.2. Advancements and Future Directions

Despite these challenges, ordered porous silica materials have shown promise due to their high drug loading capacity and controlled drug release properties. These materials have been successfully used to load various drugs and deliver them to target tissues and organs. Enhancing the loading capacity and controlling drug release can be achieved through the modification of these materials with functional groups, which warrants further development. Innovative surface modifications not only improve delivery capacity but also introduce specific therapeutic properties depending on the material’s composition. The introduction of the Al atom or other heteroatoms into the framework of ordered silica porous materials will also change their properties, e.g., the ion exchange ability and the interaction between folic acid and Al-containing ordered silica porous materials. All of these abilities are hardly obtained in the non-ordered silica materials, e.g., SiO_2_ nanoparticles. As synthesis technologies for ordered porous silica materials advance, new materials with novel textural structures and morphologies may emerge, potentially enhancing their application in drug delivery systems.

## Figures and Tables

**Table 1:** The structures of zeolites frequently used in drug delivery systems.

Codes	Connection Mode	Channels	Tiling Arrangement	Idealized Cell Data
MFI	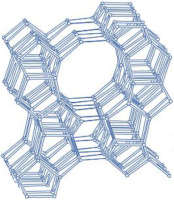	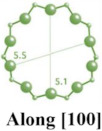 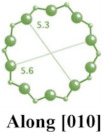	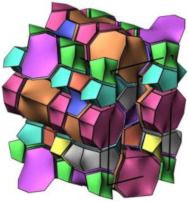	orthorhombic, Pnma, a = 20.1 Å, b = 19.7 Å, c = 13.1 Å
FAU	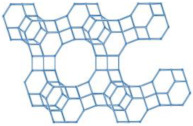	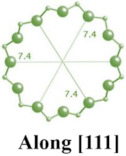	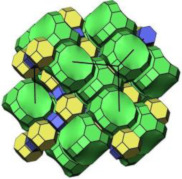	cubic, Fd$\bar{3}$m, a = 24.3 Å
LTA	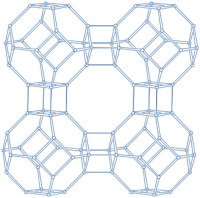	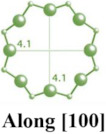	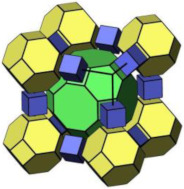	cubic, Pm$\bar{3}$m, a = 11.9 Å
HEU	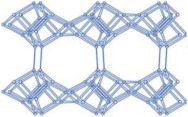	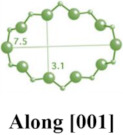 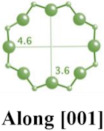 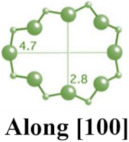	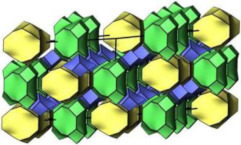	monoclinic, C2/m, a = 17.5 Å, b = 17.6 Å, c = 7.4 Å, β = 116.1°
MOR	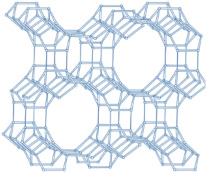	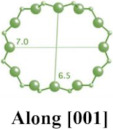 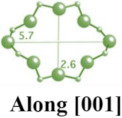	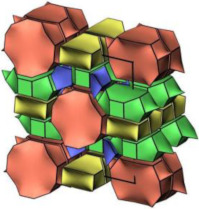	orthorhombic, Cmcm, a = 18.3 Å, b = 20.5 Å, c = 7.5 Å
CHA				

## Data Availability

Not applicable.
